# Global registry of alopecia areata disease severity and treatment safety: findings from an international pilot and optimization of a scalable real-world registry

**DOI:** 10.3389/fmed.2026.1796271

**Published:** 2026-10-14

**Authors:** Lara Cutlar, Jisha Jasmin, Blake Smith, John Bourke, Ulrike Blume-Peytavi, Laita Bokhari, Chantal Cotter, Rachita Dhurat, Ncoza Dlova, Samantha Eisman, Godfrey Fletcher, John Frewen, Matthew Harries, Alan D. Irvine, Nekma Meah, Ian McDonald, Manabu Ohyama, Yuliya Ovcharenko, Francesca Pampaloni, Bianca Maria Piraccini, Lidia Rudnicka, David Saceda-Corralo, Michela Valeria Rita Starace, Pearl Olesitse, Rodney Sinclair, Dmitri Wall

**Affiliations:** 1 National and International Skin Registries (NISR) CLG, Dublin, Ireland; 2 Charles Institute of Dermatology, School of Medicine, University College Dublin, Dublin, Ireland; 3 Sinclair Direct, East Melbourne, VIC, Australia; 4 Department of Dermatology, South Infirmary Victoria University Hospital, Cork, Ireland; 5 Department of Dermatology, Venereology and Allergology, Charité-Universitätsmedizin Berlin, Berlin, Germany; 6 Dermatology, Children’s Health Ireland, Dublin, Ireland; 7 Dermatology, LTM Medical College & Hospital Sion, Mumbai, India; 8 Dermatology Department, University of KwaZulu-Natal, Durban, South Africa; 9 Sinclair Dermatology, East Melbourne, VIC, Australia; 10 Centre for Dermatology Research, Faculty of Biology, Medicine and Health, The University of Manchester, Manchester, United Kingdom; 11 Department of Dermatology, Salford Royal Hospital, Northern Care Alliance NHS Foundation Trust, Manchester Academic Health Science Centre, Manchester, United Kingdom; 12 Clinical Medicine, Trinity College Dublin, Dublin, Ireland; 13 Mersey and West Lancashire Teaching Hospitals NHS Trust, St Helens, United Kingdom; 14 Faculty of Biology, Medicine and Health, Manchester University, Manchester, United Kingdom; 15 Department of Dermatology, Mater Misericordiae University Hospital, Dublin, Ireland; 16 Department of Dermatology, Kyorin University School of Medicine, Tokyo, Japan; 17 Department of Infectious Diseases and Clinical Immunology, V.N. Karazin Kharkiv National University, Kharkiv, Ukraine; 18 Dermatology Unit, IRCCS Azienda Ospedaliero-Universitaria di Bologna, Bologna, Italy; 19 Department of Medical and Surgical Sciences, University of Bologna, Bologna, Italy; 20 Private Practitioner, Bologna, Italy; 21 Department of Dermatology, Medical University of Warsaw, Warsaw, Poland; 22 Hair Disorders Unit, Dermatology Department, Ramon Y Cajal Hospital, University of Alcala, IRYCIS, Madrid, Spain; 23 Faculty of Medicine, University of Melbourne, Melbourne, VIC, Australia; 24 Hair Restoration Blackrock (HRBR), Dublin, Ireland

**Keywords:** alopecia areata, epidemiology, JAK inhibitors, minoxidil, patient registries, real-world data

## Abstract

**Background:**

Harmonized international registries are essential for collecting diverse, real-world data to understand and inform how evolving practice impacts disease outcomes, particularly in a rapidly changing treatment landscape, such as with alopecia areata (AA). The Global Registry of Alopecia areata disease Severity and treatment Safety (GRASS) was established following a multi-stakeholder, international, consensus exercise to establish an AA-specific dataset. Extending work conducted by an international atopic dermatitis registry network aimed to reduce data heterogeneity across country and disease borders, to optimize comparative outcome potential benefits, reduce input fatigue and resource utilization that derives from superfluity that reduces sustainability.

**Methods:**

GRASS was piloted across four countries (Ireland, Italy, Spain, and Australia) using a web-based application. Following ethics and privacy approvals, >1000 patients were recruited under an initial 354-point dataset. A subsequent comprehensive review was conducted to streamline data entry and isolate high-value research variables.

**Results:**

Recruitment remained steady, but completion rates were suboptimal: 40% of patients lacked longitudinal follow-up data. Field non-completion rates were 33.7% at baseline and 52.2% at follow-up. Consequently, the dataset was rationalized into the GRASS SEED (Short Easily Enterable Dataset) protocol. This revision reduced data points by 56% (eliminating 198 of 354 fields), significantly lowering clinical enrollment burden.

**Conclusion:**

The GRASS SEED protocol provides a sustainable template to minimize administrative burden, enhance site compliance, and generate robust real-world evidence for emerging therapeutic outcomes.

## 1 Introduction

The Global Registry of Alopecia Areata Disease Severity and Treatment Safety (GRASS) is an international initiative designed to address the critical need for comprehensive, harmonized, real-world data on alopecia areata (AA), an autoimmune condition that can cause unpredictable and often distressing hair loss that can have a marked impact on quality of life (1, 2).

The registry was developed with technology partners, following a global consensus exercise involving physicians, patient organizations, scientists, pharmaceutical industry representatives, and pharmacoeconomic experts (3). This collaborative effort identified core domains and data items essential for building a standardized network of AA patient registries, enabling the collection of comparable data on disease severity and treatment safety across diverse populations and clinical settings. This approach was designed to facilitate recording of a robust, harmonized dataset that could empower clinicians and researchers to oversee existing and emerging treatments in real-world settings at a time when the therapeutic landscape for AA is rapidly evolving. To further build on the capacity for collaboration, the framework for GRASS was derived from successful models and datasets from other dermatology registries, such as the TREAT Registry Taskforce for atopic dermatitis, with a view to promoting data harmonization and cross-disease comparisons. This approach was expected to create opportunities to build and validate new tools, such as those to assess the health-related quality of life in AA patients, ensuring that patient-centered outcomes are integrated into the registry's data collection and analysis (4). The network-building capacity is also hoped to facilitate new research opportunities and borrow from the diverse collective experience of the involved communities to turn data into the knowledge and wisdom.

This paper describes the implementation of GRASS International and summarizes the outcomes of data collection in its pilot phase. We present the lessons learned that prompted the evolution of GRASS SEED (Short Easily Enterable Dataset), which will be key to facilitating the accelerated and sustainable global implementation of this registry beyond the over 1000 patients already enrolled.

We hope that GRASS will represent a transformative step toward global collaboration in AA research, offering a scalable, harmonized platform for monitoring disease severity, treatment safety, and patient quality of life. With ongoing evolution, we hope that it will help shape the future of AA management and research worldwide, offer an established platform through which new and innovative technologies can be introduced, and, as a multinational registry, that it could be instructive in the establishment of new, and connection of existing, patient registries.

### 1.1 Alopecia areata

Alopecia areata (AA), an autoimmune-mediated disorder, characterized by nonscarring hair loss, ranging from a single patch to multifocal patches, diffuse or complete hair loss of the scalp with or without involvement of other hair bearing areas (5, 6). While the definition of severity of AA is evolving, with increasing awareness of necessity to acknowledge site involvement (e.g., eyebrows or eyelashes, or cosmetically difficult to camouflage areas on the scalp), rate of loss, impact on quality of life and treatment resistance as part of compound severity assessments, scalp area involved has been the traditionally utilized surrogate measure of severity for clinical practice and in randomized controlled trials (1, 2, 7, 8). The Severity of Alopecia Tool (SALT) represents what % of the scalp has been involved (9, 10). While debate exists regarding what % constitutes mild, moderate and severe disease, there is broad agreement regarding the definitions posed by the Alopecia Areata Investigator Global Assessment (AAIGA) scale and Alopecia Areata Scale (AAS) which both define mild (or limited) and moderate disease as ≤20 and 21%–49% respectively (7, 11). While both recognize severe disease as greater than 50% scalp involvement, the AAIGA adds a very severe category for those with ≥95% scalp involvement.

Although multiple factors, such as hormonal, psychological or physical stressors, concomitant illnesses or even vaccines (12) have been theorized as precipitating factors in genetically susceptible individuals, the exact etiology is yet to be ascertained (13, 14). Population incidence of AA is estimated to be 1–2 per 1,000 people (15–17), with a projected rate of 1,550,600 new incidences globally, each year (2). Spontaneous regrowth occurs in approximately 67% of patients with single or multifocal patchy disease within approximately 12 months (6, 18). The remaining patients typically progress to either severe alopecia areata in 45% (total (30%), with full scalp loss and universalis (15%), when all body hair is lost, or develop a remitting-relapsing course in 55%, with multiple episodes throughout their lifespan (19, 20). In individuals with severe disease, spontaneous remission is rare, with recent clinical trials suggesting a placebo response rate between 0.8 and 6.2% in trials that recruited patients with SALT ≥50 (21, 22).

AA is associated with atopic dermatitis (AD), with recent research showing that those with more severe AA appear to have a higher incidence of AD, of which there is a greater prevalence of moderate to severe disease (23). Patients with AA also have increased autoimmune comorbidity, including vitiligo, hypothyroidism and particularly systemic lupus erythematosus. The association of AA and cardiovascular disease is an important, but contentious consideration in the context of conflicting evidence about whether a true association exists (24, 25). Indeed, JAK inhibition, emerging as a breakthrough AA therapy, is itself a subject of conflicting evidence, both with respect to cardiovascular disease and thromboembolic disease. However, emerging clinical trial and real-world evidence suggests a more reassuring safety profile in the context of the treatment of AD and AA compared with rheumatoid arthritis, in which concerns were initially raised (26–28).

For those with AA who are severely affected, its chronic, relapsing course can cause significant psychosocial morbidity, including distress, sleep disturbance, anxiety, depression, and suicidality (29, 30). Patients with AA are also more likely to demonstrate absenteeism or presenteeism and be prescribed antidepressants than the general population (31). Regarding out of pocket expenses associated with AA, research in the UK identified that, of services and products utilized by people with AA, the annual median total cost was £801.50 (IQR £206–1946.55). There was a marked disparity between women, £930 (IQR £300–£2125.40) for whom wigs were the most used and had a median annual cost of £700, compared to men (Median £155 (IQR £18.75–£696.80)), for whom private dermatology services represented the biggest individual expense (£550 annually), though only 14 of 107 men studied had utilized private services. On average, individuals allocated approximately 3% of their disposable income (before housing expenses) to products and services related to AA, with women's median cost again substantially higher (3.6% vs. 0.3%) higher than men's (32).

Though traditional therapies, including systemic immunomodulatory treatments have been disappointing, new standards have been set by the emergence of JAK inhibitions, firstly in real-world evidence (33–44) and more recently, industry-sponsored, randomized controlled trials (45–47). These have been instrumental in the development and regulatory approval of baricitinib, ritlecitinib, deuruxolitinib and most recently upadacitinib (48), as the first licensed systemic therapies for AA. Though effective, suboptimal or poor response in a significant proportion of patients, and limited availability in many countries, means that non-approved systemic immunomodulatory, intralesional and topical therapies alone, or in combination therapy remain a necessity in real-world settings. The burden of therapy and disease can therefore remain elevated, and require adjunctive measures, for example the use of hairpieces, application of sunscreen, camouflage techniques such as hair fibers and micropigmentation, and ocular protection if eyelashes are absent, further impacting on quality of life.

The burden of alopecia areata (AA) therefore extends far beyond hair loss, imposing significant personal and economic costs on affected individuals and healthcare systems globally. These costs are driven by the chronic nature of the disease, the need for sustained treatment, and the deep psychosocial impact of visible hair loss. The personal cost of AA is defined by significant psychosocial morbidity, including profound distress, anxiety, and depression that can severely impact self-esteem, quality of life, and social functioning, driving a high willingness to pay for effective treatment and cosmetic solutions. The financial burden is likely to increase further due to the licensing of JAK inhibitors. An Australian group calculated cost per ‘cure’ (a reduction to SALT ≤ 20) for baricitinib to be AUD $ 2695.87 (49). For ritlecitinib, (UK National Health Service costing) it is approximately AUD $ 2693.87 per month (49). The financial burden for payers is likely to increase further due to the licensing of JAK inhibitors, with a cost per ‘cure’ (based on a reduction of scalp area affected to ≤20) of baricitinib in Australia of AUD$2695.87 and ritlecitinib, based on UK National Health Service costing, at approximately AUD$2693.87 per month (49).

With significant financial ramifications for health services, real-world evidence will be required to aid pharmacoeconomic analyses of the impact of novel therapies compared to existing therapies and prostheses and guide reimbursement thresholds for payers. This will need to be balanced with indirect societal cost which includes significant productivity loss (43) and disability claims, though, unlike the costs of medicines, these costs are more difficult to calculate precisely. When compared to similar inflammatory conditions like AD, AA is shown to impose a high economic burden, highlighting the need for better access to affordable and effective management strategies. Real-world evidence will also be required to assess the long-term safety of emerging treatments, particularly where growing evidence suggests that a the great majority of patients who respond well to JAK inhibitors, will require long-term therapy.

The breadth of issues outlined above illustrates why the development of a meaningful AA registry is inherently challenging. To generate clinically useful real-world evidence, the registry needed to capture not only disease severity, treatment exposure and outcomes, but also comorbidities, quality of life, safety, health-economic data, other disease-specific variables, and data harmonization with related disease registries. At the same time, it had to remain sufficiently practical to achieve high-quality, complete data collection in routine clinical practice. Understanding this balance between comprehensiveness and feasibility is fundamental to understanding the design, evolution and eventual simplification of the GRASS registry.

## 2 Methods

In 2021 a multi-stakeholder, international consensus was published to establish core domains and data items of the registry, and GRASS was piloted in 5 sites, (Australia, Italy and 3 in Ireland), to assess suitability in diverse, real-world settings (3). Entry is performed by trained registry data collectors who input information derived from clinical encounters and medical charts, ensuring a consistent clinical coding scheme across all sites. Data, derived from provision of routine clinical care, was collected from consented patients’ charts, without the need for any registry-only visits. Inclusion criteria were a clinical diagnosis of AA affecting the scalp or other sites (e.g., including eyelashes, eyebrows and beard); written informed consent, (assent if under 18 years of age); willingness to complete registry-related questionnaires. Exclusion criteria included an absence of a clinical diagnosis of AA, or inability to provide written, informed consent.

Patients’ first visit to the clinic was recorded as a ‘Baseline’ and all others as ‘Follow Up’ visits. SALT scores were recorded for all timepoints captured in a patient's chart. The GRASS registry utilizes a centralized, cloud-based proprietary clinical research platform designed for multi-center longitudinal studies. To ensure data security and regulatory compliance across international borders, the architecture employs encryption and is hosted on servers compliant with GDPR (General Data Protection Regulation) and HIPAA standards. The data is hosted in an ISO 27001 certified data center located in Frankfurt, Germany. Data entry was performed by the clinic staff, or research office where available. The same consent and information leaflet template was used to ensure harmonization across sites and a protocol document was provided to train the clinic team on data entry and quality control procedures.

The ownership of an individual's data remains with that patient, while NISR Solutions CLG and Sinclair Dermatology maintains governance. Access for research is only granted after meeting strict ethical and safety criteria and with the approval of the GRASS executive committee. Cooperation across borders was facilitated through a federated model. This allowed individual countries (e.g., GRASS-Ireland, GRASS-Italy, GRASS-Australia) to maintain sovereignty over their local data while utilizing a standardized global dataset to ensure international interoperability. Participating sites maintain control over their respective cohorts through formal Data Transfer Agreements (DTAs), and all data use is subject to approval by an Executive Committee and relevant Research Ethics Committees (REC).

## 3 Results

With first implementation in Australia, beginning in March 2022, data from this pilot represents 4 years of data acquisition. However, baseline data collection also incorporated retrospective patient data, resulting in data representing more than 4 years for 28.2% of patients (Figure 1). The majority of patients (47%) have 3–4 years of data recorded. Almost 60% of patients have a Follow up visit recorded, Figure 2. Figure 3, a heatmap of data completion rates, identifies the percentage of fields completed in the Baseline visit and the across all the Follow up visits. At Baseline, 33.7% of data fields were incomplete, compared with 52.2% for the follow up visits.

**Figure 1:**
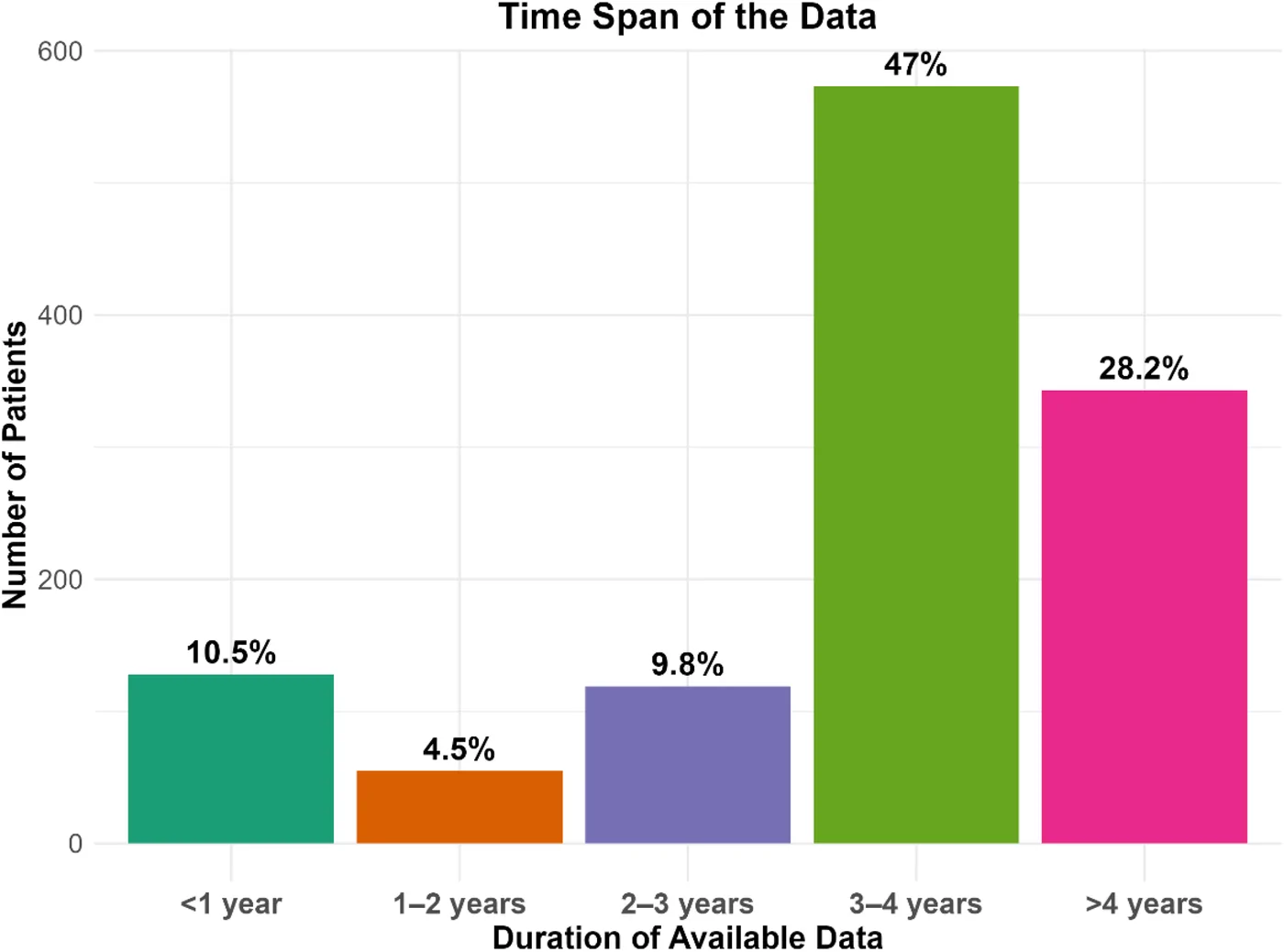
Years of data collected by number of patients. Data duration was defined as the interval between the earliest available clinical record and the analysis cut-off date.

**Figure 2:**
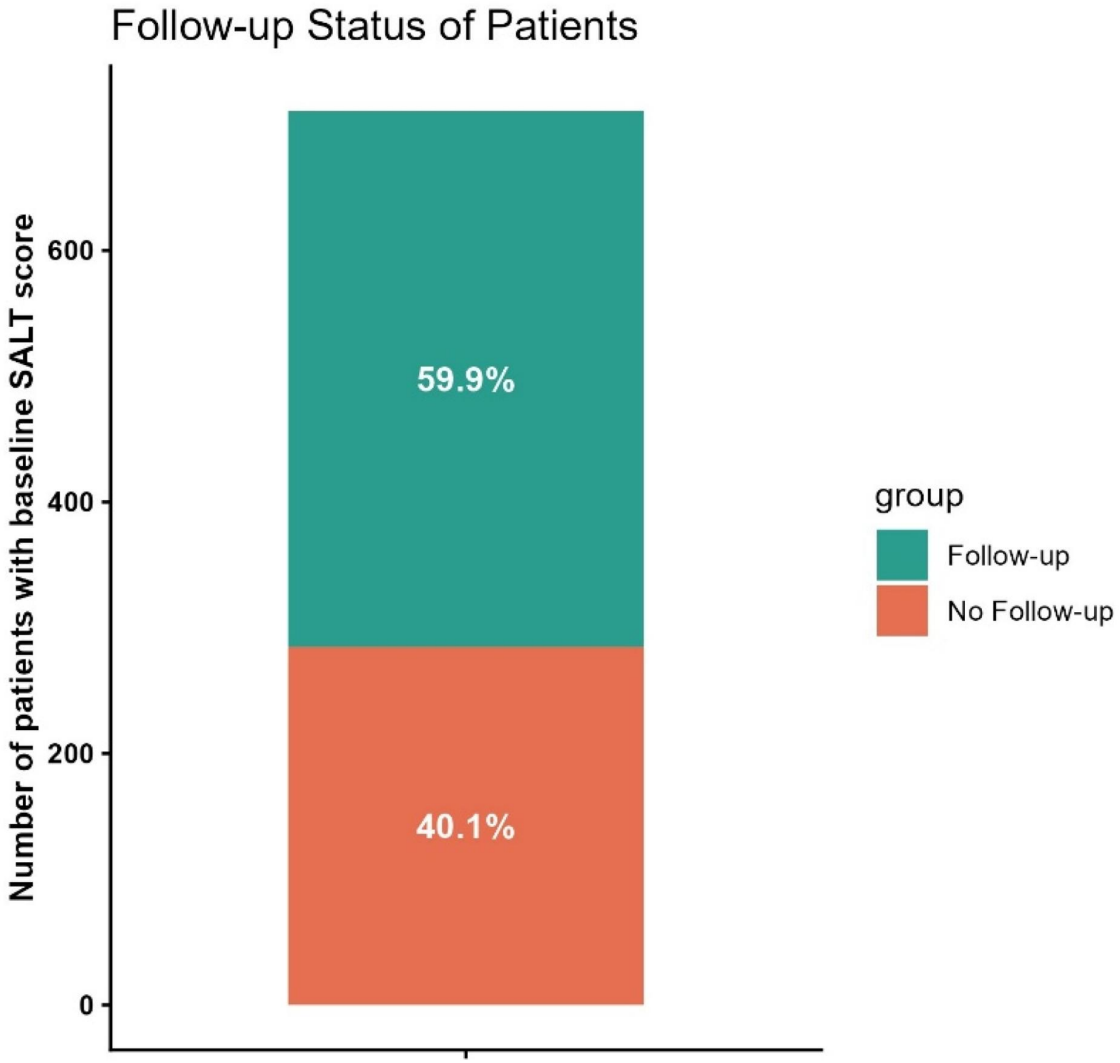
Follow up status of GRASS patients with a baseline SALT score recorded. Stacked bar chart showing the proportion of patients with and without follow-up SALT assessments. Of the 711 patients included, 426 (59.9%) had at least one follow-up assessment beyond baseline, while 285 (40.1%) had only a single recorded visit. Percentages are calculated relative to the total cohort.

**Figure 3:**
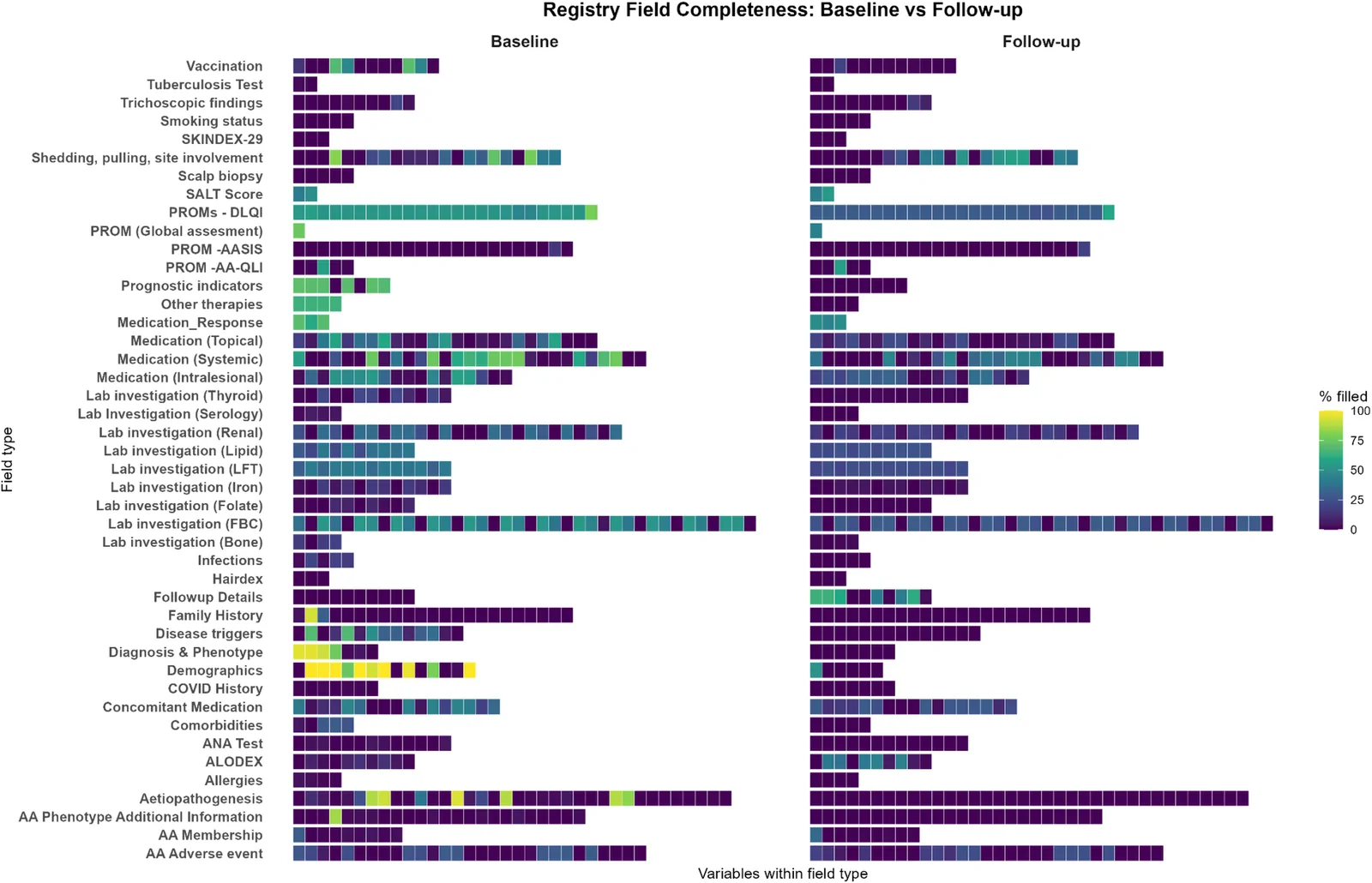
Heatmap of registry field completeness of GRASS registry from pilot data. Heatmaps showing the percentage completeness of individual data fields within each registry field type at baseline (left) and follow-up (right). Each tile represents a single variable within the field type (*y*-axis), with colour intensity corresponding to the proportion of registered patients for whom data were available for that variable. Completeness was calculated as the percentage of patients with recorded data for a given variable relative to the total number of patients in the registry at the corresponding time point.

Table 1 provides an overview of longitudinal data availability across major clinical, treatment, laboratory, and patient-reported outcome (PROM) domains within the registry.

**Table 1:** Summary of follow-up data completeness by registry field type.

Field type	Follow-up completeness pattern	Approximate completeness (%)	Key observation
Follow-up details	High	42–64	Visit type, visit schedule, date of follow-up among the most complete variables
Disease severity (SALT, AA Global assessment)	High	42–54	Core disease monitoring outcomes consistently recorded
Site involvement, hair characteristics	High	39–56	Site involvement, pigmentation and wash day assessment are frequently completed
PROM-DLQI	Moderate to High	25–59	Individual questions and score generally captured
PROM AAQLI	High	∼57%	Questionnaire completed and scores are recorded
PROM-AASIS	Low	0–20	Sparse item level follow-up data
Systemic medications	Moderate to High	33–45	Treatment exposures, effectiveness, duration and adverse events monitoring are routinely recorded
Intralesional medications	Moderate	20–37	Treatment administration & response recorded
Topical medications	Moderate	17–23	Exposure and administration variables recorded, detailed descriptors less complete
Adverse events	Low to Moderate	0–28	Some adverse event monitoring captured, detailed attribution fields often missing
Full Blood Count (FBC)	Moderate	25–29	Most consistently completed laboratory domain
Liver Function Test	Moderate	20–24	Routine monitoring variables recorded
Lipid Profile	Moderate	22–26	Selective longitudinal data capture
Renal investigation	Low to Moderate	5–20	Some monitoring variables recorded, with many low completions
Thyroid investigation	Low	0–2	Infrequently repeated at follow-up
Iron studies	Low	0–4	Limited follow-up
Folate/B12	Low	∼0	Rarely repeated
Vaccinations	Low	0–19	Other than COVID vaccination, primarily a baseline historical information
Smoking history, Disease triggers,	Low	0	Baseline information
Allergy assessments, comorbidities, Biopsy variables	Low	0–10	Mostly baseline information, rarely updated

The table provides an overview of longitudinal data availability across major clinical, treatment, laboratory, and patient-reported outcome (PROM) domains within the registry.

A substantial number of variables showed no follow-up completion despite being recorded at baseline. These were predominantly historical or descriptive variables not typically expected to change over time. Examples include detailed diagnostic and phenotypic descriptors (e.g., clinical diagnosis, histopathology, alopecia subtype classifications, autoimmune disease subtypes, allergy subtypes, disease trigger classifications, smoking history details, family history subcategories, and comorbidity details). Similarly, several vaccination variables (vaccination status, vaccination date, and vaccination type), allergy testing outcomes, serology and infection-related variables, biopsy characteristics, and supplementary phenotype descriptors demonstrated 0% follow-up completion. Many item-level responses within the Alopecia Areata Symptom Impact Scale (AASIS) questionnaires were also absent at follow-up.

Variables with low follow-up completeness were primarily related to detailed laboratory investigations and treatment metadata. This included thyroid function tests (TSH, T3, T4), folate and vitamin B12 investigations, iron studies (serum iron, ferritin, transferrin, TIBC), renal function measurements (creatinine, urea, electrolytes, bicarbonate, eGFR), serology and tuberculosis investigations, bone investigations, scalp biopsy details, and infection-related variables. Detailed treatment fields such as dose adjustments, treatment cessation reasons, concentrations, administration details, and adverse-event attribution also demonstrated relatively low completion. Follow-up percentages in these domains were generally below 20%, with many variables ranging between 0%–10%.

Moderate follow-up completeness was observed for a range of treatment-monitoring and laboratory variables. Full blood count (FBC) parameters, liver function tests (LFTs), lipid profiles, concomitant medication records, topical treatment variables, intralesional treatment variables, and selected renal monitoring variables frequently demonstrated follow-up completion between 20% and 40%. Examples included hemoglobin, leukocyte, platelet and differential blood counts (approximately 25%–30%), liver enzymes and albumin (approximately 20%–24%), lipid measurements (approximately 22%–26%), and medication exposure variables including treatment duration, frequency, route, dose coding, and treatment effect (approximately 20%–45%).

The highest follow-up completeness was observed for variables directly related to disease monitoring, treatment response, and clinical follow-up encounters. Follow-up visit variables demonstrated particularly high completeness, including visit type (61.3%), visit scheduling (42.3%), cohort status (63.8%), age at follow-up (63.8%), and overall follow-up change assessments (41.1%). Levels of completeness varied for: SALT score variables (42%–53%), hair pigmentation assessments (52%), site involvement assessments (56%), wash-day variables (39%–43%), AA Global Assessment (43.6%), and treatment response outcomes such as follow-up treatment, treatment response, and hair growth (44%–48%).

PROMs demonstrated variable completeness. AA-QLI showed relatively high follow-up completion, with questionnaire completion rates of approximately 56%–57%. DLQI demonstrated moderate-to-high follow-up completeness, with individual question responses and total scores generally ranging from 25%–31%. In contrast AASIS primarily showed evidence of questionnaire administration rather than complete capture of item-level responses, with most individual questions demonstrating minimal or no follow-up completion.

Medication-related domains were among the most comprehensively recorded longitudinally. Systemic therapy variables showed follow-up completion rates of approximately 33%–45% for treatment continuation, frequency, route, medication exposure, effectiveness, and adverse-event severity. Similarly, intralesional therapy variables demonstrated follow-up completeness of approximately 20%–37% for treatment site, concentration, volume, duration, frequency, and response. Topical therapy variables showed follow-up completion ranging from approximately 17%–23% for treatment exposure and administration characteristics, with lower completion for detailed treatment-specific descriptors.

A total of 1,128 patients were enrolled across three regions: Australia (853), Ireland (171), Spain (1) and Italy (193). The mean age at the time of consent was 35.9 ± 16.3 years, with females comprising 36.9% of the cohort. The demographic distribution included 85.9% adults, 8.5% adolescents, and 5.6% children. Ethnicity was recorded as predominantly White (66.6%) with South Asian being the next highest represented. Further details reported in Table 2.

**Table 2:** Baseline demographic and clinical characteristics of patients enrolled in the GRASS registry, stratified by country.

Variable		Total (*N* = 1,128)
Age (years), mean ± SD		35.9 ± 16.3
Age group, *n* (%)	Child (0–11 years)	68 (5.6%)
	Adolescent (12–17 years)	104 (8.5%)
	Adults (≥18 years)	1046 (85.9%)
Sex, *n* (%)	Male	768 (63.1%)
	Female	450 (36.9%)
Ethnicity, *n* (%)	White	810 (66.6%)
	South-Asian	101 (8.3%)
	Asian-Chinese	43 (3.5%)
	Asian-Others	21 (1.7%)
	Black-African	11 (0.9%)
	Hispanic-Latino	6 (0.5%)
	Others	38 (3.1%)
	Not available	188 (15.4%)
SALT score (baseline)	Reported	711
	Not reported	417
	Mean ± SD	26.2 ± 36.6
	Median (IQR)	5 (1–48)
SALT score, *n* (%)	Score ≤20	480 (67.5%)
	Score 21–49	54 (7.6%)
	Score 50–94	76 (10.7%)
	Score ≥95	101 (14.2%)

Data are presented as mean ± standard deviation (SD) for continuous variables and number (percentage) for categorical variables. Percentages are calculated within each column. Age is reported at the time of consent. Age groups are defined as children (0–11 years), adolescents (12–17 years) and adults (≥ 18 years). Ethnicity categories are reported as recorded in the registry. ‘Not available’ denotes missing or unreported ethnicity data. Disease severity is assessed using the Severity of Alopecia Tool (SALT). SALT scores range from 0 to 100, with higher scores indicating greater scalp hair loss. SALT scores were derived using baseline measurements where available. Summary statistics for SALT scores are presented as mean ± SD and median (interquartile range [IQR]). SALT score categories are defined as ≤20 (mild), 21–49 (moderate), 50–94 (severe), and ≥95 (very severe).

SD, standard deviation; IQR, interquartile range; SALT, Severity of Alopecia Tool.

Baseline Severity of Alopecia Tool (SALT) scores were documented for 711 subjects (63.0%) and omitted/not reported for 417 subjects (37.0%). Most of the documented population (67.5%) falls within the lowest severity tier (<20). Conversely, the remaining population is distributed non-linearly across higher severity bands, with a secondary minor concentration (14.2%) at the absolute highest severity threshold (> 95).

Comorbidity data was reported in over 20% of cases Table 3 for at least 1 autoimmune comorbidity. A summary of the numbers of patients by medication treatment shows the predominant data collection was for treatment with oral minoxidil, followed by corticosteroids in Table 4. Various JAK Inhibitors (JAKi) are recorded in the registry to date; baricitinib, tofacitinib, ritlecitinib, upadacitinib and ruxolitinib. In total 67% of patients on the registry had or are being treated with a JAKi.

**Table 3:** Distribution of comorbidities among patients enrolled in the GRASS registry (*N* = 1,128), stratified by country.

Comorbidity	Total, *n* (%)
Patients with ≥1 autoimmune comorbidity	239 (21.2%)
Thyroid issue	102 (9%)
Psoriasis	38 (3.4%)
Vitiligo	27 (2.4%)
Coeliac disease	16 (1.4%)
Rheumatoid arthritis	13 (1.2%)
Type 1 diabetes	10 (0.9%)
Other autoimmune condition	76 (6.7%)
Allergic rhinoconjunctivitis	177 (15.7%)
Asthma	207 (18.4%)
Atopic dermatitis	252 (22.3%)
Other Allergic conditions	11 (1%)
Patients with ≥1 other kinds of comorbidity reported	313 (27.7%)
Anxiety Disorder	31 (2.7%)
Pure Hypercholesterolaemia	23 (2%)
Depressive episode	21 (1.9%)
Iron deficiency	18 (1.8%)
Gastro-oesophageal reflux	17 (1.5%)
Endometriosis	12 (1.1%)
Acne	10 (0.9%)
Migraine	10 (0.9%)
Irritable bowel syndrome	9 (0.8%)
Osteoporosis	9 (0.8%)
Attention deficit	8 (0.7%)
Insomnia	7 (0.6%)
Polycystic ovarian syndrome	6 (0.5%)
Other reported comorbidities	261 (24.7%)

Data are presented as number (percentage) of patients. Percentages are calculated within each column using the total number of patients in that column as the denominator. Comorbidities are grouped into autoimmune, allergic, and other categories. Individual comorbidities are reported separately; patients may have more than one condition within or across categories, and therefore counts and percentages are not mutually exclusive and may not sum to the total number of patients. ‘Other autoimmune condition,’ ‘Other allergic conditions,’ and ‘Other reported comorbidities’ include conditions not listed individually. No comorbidities were reported in patients from Spain; therefore, this country is not presented in this table.

**Table 4:** Distribution of patients with alopecia areata across age groups across target systemic therapies.

Medication	No. of patients	Children (Age 0–11 years)	Adolescents (Age 12–17 years)	Adults (Age 18+)
Oral Minoxidil	813	56	116	641
Corticosteroids	536	53	9	394
JAK inhibitors (Baricitinib, Tofacitinib, Ritlecitinib, Upadacitinib, Ruxolitinib)	654	36	114	504
Traditional immunosuppressants (Azathioprine, Ciclosporin, Methotrexate)	168	13	38	116

This table displays patient headcounts within each specific medication category. Age groups are defined based on the patient's age during their treatment timeline: Children (Ages 0 to 11), Adolescents (Ages 12 to 17), and Adults (Ages 18 and older). Patients who transitioned into a new age category during a continuous treatment course are counted in both respective age groups.

## 4 Discussion

The development and implementation of GRASS, in a pilot form, has demonstrated useful insights into patient populations presenting in real-world, compared to clinical trial settings. In addition to demonstrating real-world adoption of emerging JAKi therapies, it points to opportunities to understand complex treatment regimens and how they might alter response for patients who would not qualify for clinical trials, or where there is suboptimal response. The timespan of data collected, and particularly SALT scores, is just approaching a critical threshold that will enable these analyses, while also addressing the questions of treatment durability and JAKi withdrawal. To further improve the capacity of the registry to achieve these goals, however, several limitations were identified that are being addressed with changes in data structure and depth, in addition to generalizability.

We identified a significantly different population to those recruited for clinical trials. This highlights the capacity of the registry to capture the experience of a group more representative of that seen in clinical practice.

The GRASS pilot enabled a maximum data capture of 354 fields in total, Figure 4. While such an extensive dataset was acknowledged to impact data completeness, the value of supporting the broad needs of the diverse community that the registry was designed to serve was deemed more of a priority. Lower data completion than might be expected in an observational clinical trial, was not considered a critical weakness as the registry was also imagined as a potential tool to discover data-collection challenges that could be potentially overcome with the introduction of technology that would strengthen the value of the technology to collaborators. Furthermore, beginning with a maximal dataset, which could later be reduced based on real-world use and value, was considered a more appealing means of developing a core/minimal dataset than pressuring potential contributors to make concessions without the experience of real-world use at the early phase of dataset design. An additional benefit with this approach was the establishment and testing of a more and now harmonized, comprehensive dataset, that remains available for centres with greater resources to continue to collect.

**Figure 4:**
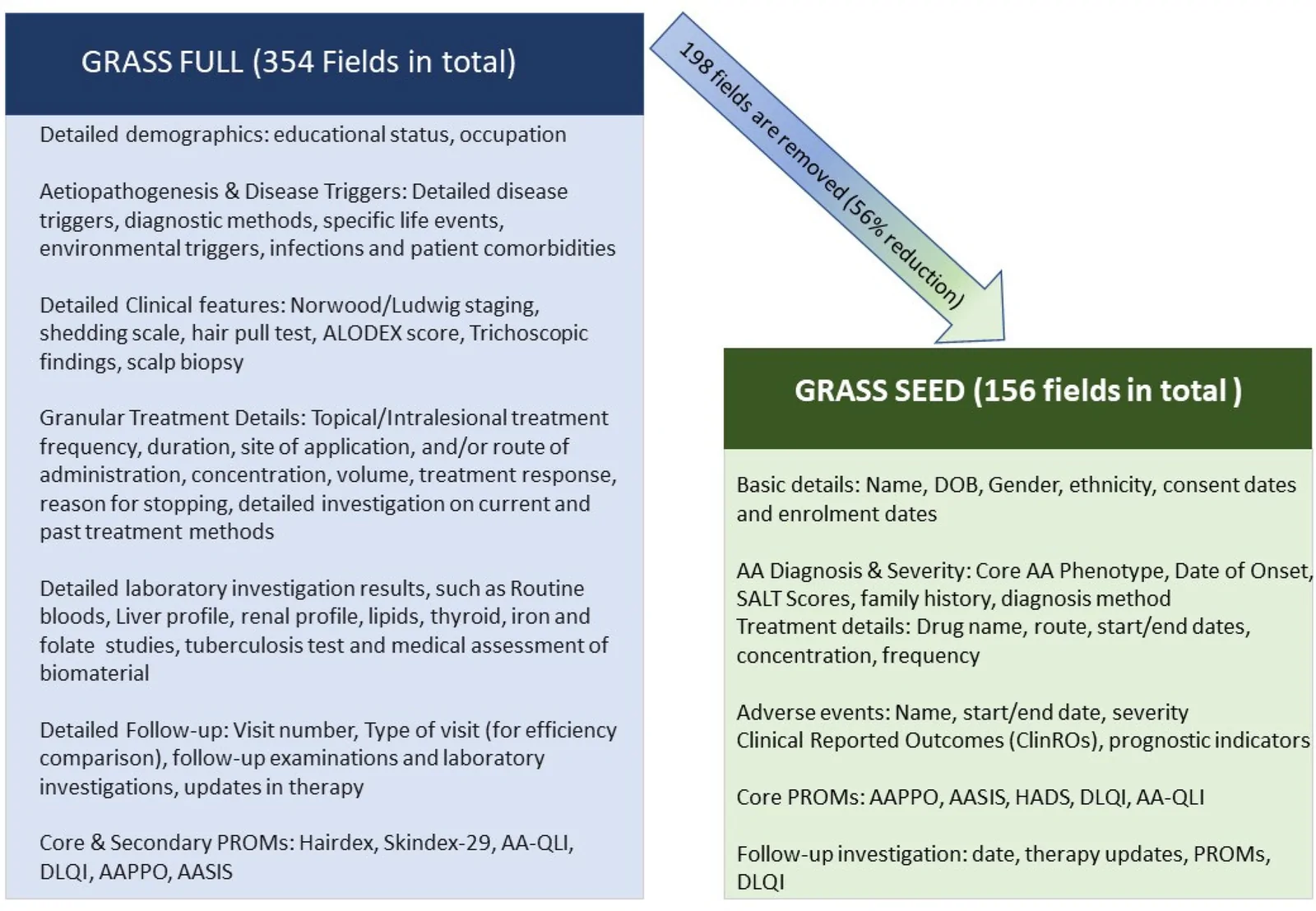
GRASS full reduction of fields to GRASS SEED.

Implementation of the initial maximal dataset in the pilot phase, as expected, created significant administrative burden for participating sites, leading to reduced efficiency and data completeness. For example SALT Baseline score completion (63%) rates were affected by sites not having a tool to assist in this data capture in a quick standardized way. With a view to adapting and condensing the dataset and platform further to increase user acceptability and identify strategies that might reduce resource requirements to improve sustainability of data collection and long-term registry durability, the concept of GRASS SEED (Short Easily Enterable Dataset) has been developed. This platform reduces the number of data fields to 156, following the withdrawal of pilot data points that were infrequently utilized or contributed minimal immediate value. It also largely eliminates free-text input options and reduces data collection requirements in repetitive forms and fields that are infrequently queried (e.g., removal of routine blood tests that would traditionally necessitate the entry of 38 individual data points). GRASS SEED is expected to streamline patient onboarding (Figure 5) by substantially reducing physician and patient fatigue and markedly increase time efficiency for data entry. Once a center has proven capacity for consistent data entry and recruitment, the complete dataset can be made accessible on the registry platform to facilitate the collection of more detailed data points.

**Figure 5:**
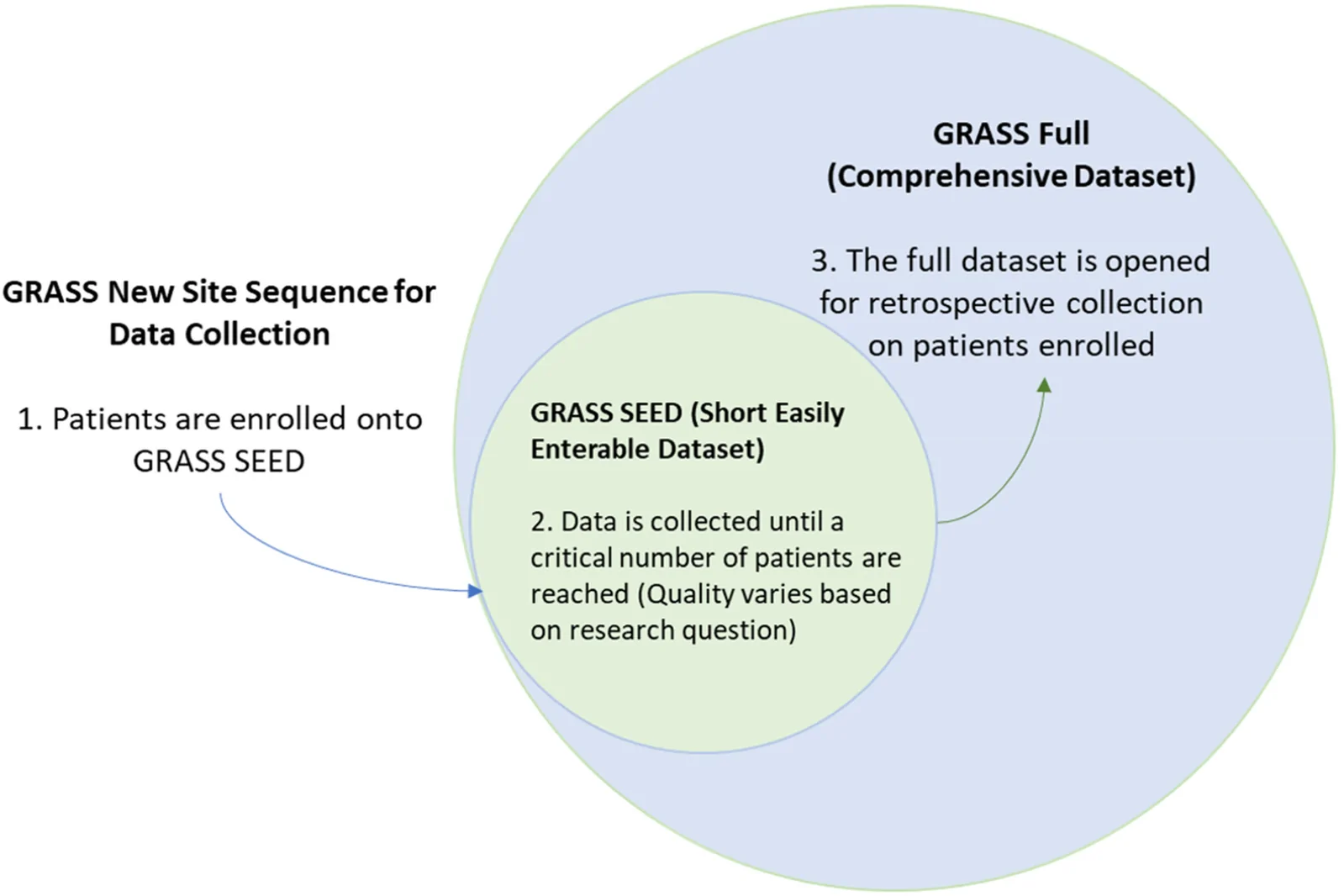
GRASS SEED comparison to GRASS full.

### 4.1 Limitations of the GRASS registry

To address emerging evidence that has demonstrated the need for additional metrics to more comprehensibly capture true disease impact than SALT score alone captures (1, 4), GRASS incorporates a number of Quality of Life metrics. There are multiple general tools which can be captured, including Dermatology Life Quality Index (DLQI) (50), Skindex-16 (51), the Women's Androgenetic Alopecia Quality of Life (WAA-QOL) (52), (AASIS) (53), Alopecia Areata Patient Priority Outcomes (AAPPO) (54), The hospital anxiety and depression scale (HADS) (55), AA-QLI (56), and the Patient Global Assessment (57). While helpful, the pilot phase noted considerable patient and physician fatigue in capturing a large range of questionnaire-based scores, in addition to logistical difficulties in administering them during routine patient encounters. To address this, the GRASS network is examining both the capacity to create and validate compound scores, as well as digital means to administer them.

While the registry has enabled the development of validated tools for assessing quality of life, there is still a need for broader integration of patient-reported outcomes and experiences, especially from underrepresented groups.

The skewed demographics of the GRASS platform in some instances (such as age and gender), represent disease characteristics, in addition to patient-likelihood to present to tertiary care. The registry, however, currently reflects limited ethnic diversity, with a strong over-representation of Caucasian participants. Addressing this limitation is crucial, and the focus on expanding to new, ethnically diverse international sites is a key goal to ensure the generalizability of future findings. A secondary aim of GRASS SEED is to reduce the resources required to extend the GRASS network in a manner that will facilitate this.

While the SEED model prioritizes efficiency, it may require a selective approach to data extraction (e.g., assigning cohorts for detailed blood work submission) to ensure critical safety and outcome data are captured without overwhelming participating clinicians.

The complexity of navigating cross-border registries demands significant resource requirements. In that regard, Figure 6 highlights countries that are at various stages of joining the GRASS network, with 4 actively recruiting currently and 15 in the process of launching. To facilitate such expansion has not just required platform adaptations with respect to language translation, and legal input to address the ever-increasing necessity to adhere to data protection legislation in multiple jurisdictions, but also the formation of the GRASS International Charity Organization, to act as a legal entity that can act as umbrella entity to link each sovereign registry that forms the GRASS network.

**Figure 6:**
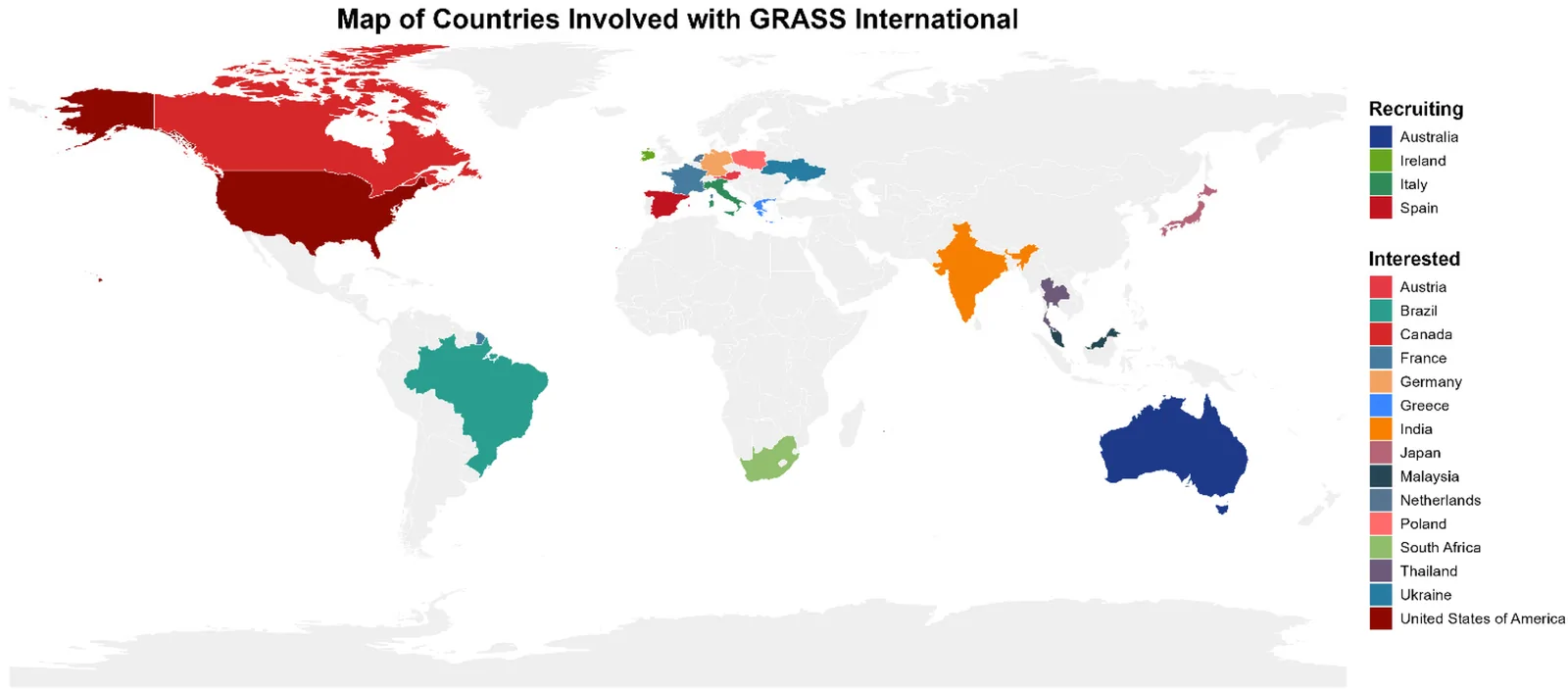
Map of countries involved with GRASS international.

While the GRASS network remains in its infancy with respect to longitudinal data, the capacity to support collaborative efforts has already borne fruit, with a number of associated projects delivered by the extended GRASS community (20, 58–61) focusing on best practices for diagnosis, laboratory evaluation and treatment of AA.

### 4.2 Lessons for future registries

#### 4.2.1 Build a community, rather than a database

Though the skillsets and experience required to build an appropriately high-quality platform is considerable, the critical driver of registry success is the supportive community that recognizes its purpose and provides the intense and persistent resourcing necessitated over extended time periods.

#### 4.2.2 Standardization, then minimization

The use of an international consensus such as an eDelphi exercise, can generate a universally relevant dataset. Real-world pilot use can then facilitate the establishment of a more core dataset based on use, value and resource requirements.

#### 4.2.3 Federated sovereignty

Ensuring local governance and ownership (e.g., GRASS-Ireland), while sharing a common platform, can build collaborative capacity, while reducing cost and some of the legal complexity of international data sharing.

#### 4.2.4 Stewardship over ownership

Positioning the registry as a “steward” rather than an “owner” of data builds higher trust with patient advocacy groups and aligns with data protection standards.

### 4.3 Conclusion

There is a recognized paucity of real-world, comparable data on the effectiveness and safety of AA therapies, a gap the GRASS registry directly addresses by collecting comprehensive, harmonized data. While this paper focuses on infrastructure and design, subsequent publications will utilize the GRASS data to perform focused analyses regarding treatment-specific outcomes, including dose-response relationships and long-term safety profiles.

The current GRASS dataset (*N* > 1,000) represents a robust source of real-world evidence reflecting contemporary AA management across three continents. This is underscored by the high signal for therapeutic diversity, particularly the inclusion of 654 unique patients receiving JAK inhibitors, such as ritlecitinib (Table 4), positioning GRASS as a vital platform for pharmacovigilance and comparative effectiveness research of novel systemic treatments. The capability for longitudinal follow-up is essential for monitoring the chronic and relapsing nature of AA and understanding long-term safety profiles.

The systematic evolution of GRASS underscores the critical balance between comprehensive data collection and operational sustainability in complex international registries. Our findings demonstrate that prioritizing a streamlined set of core data elements, such as seen with GRASS SEED, can overcome the administrative barriers that frequently compromise the longevity of large-scale disease registries. Until reliable automated systems are widely available, adaptive approaches like this are essential to facilitate data collection for harmonized international registries in diverse clinical settings. This transformative step establishes a scalable, harmonized, and operationally efficient infrastructure poised to inform clinical guidelines, policy, and future therapeutic development, but may itself, offer a common platform with which to introduce further technical innovations that may improve data capture and registry translation into real-world practice. Future work includes expanding GRASS to additional countries and commencing comparative effectiveness analyses.

## Data Availability

The raw data supporting the conclusions of this article will be made available by the authors, and in an anonymous aggregated form only.
